# The impact of internet health information seeking on COVID-19 vaccination behavior in China

**DOI:** 10.1186/s12889-024-17638-3

**Published:** 2024-01-04

**Authors:** Yunyun Zhang, Lidong Zhang, Hongyu Guan, Rong Hao, Wenting Liu

**Affiliations:** 1 College of Economics, Xi’an University of Finance and Economics, 710100 Xi’an, China; 2 Center for Experimental Economics in Education, Shaanxi Normal University, No. 620 West Chang’an Street, Chang’an District, Shaanxi Province, 710119 Xian, China

**Keywords:** Internet health information seeking, COVID-19 vaccination, Health behavior, Information

## Abstract

**Background:**

Amid the COVID-19 pandemic, the significance of vaccination has been emphatically underscored. As the foremost and pivotal measure for preventing COVID-19 transmission, the COVID-19 pneumonia vaccine plays an instrumental role in the global response to this infectious respiratory disease. However, COVID-19 vaccination coverage remains challenging in low- and middle-income countries and regions. Studies have found that frequent seeking to health information is also associated with healthier behaviors, but these studies have not yet focused on the impact of Internet health information seeking on individual vaccination and the mechanism of this effect.

**Methods:**

Data were obtained from the Chinese General Social Survey (CGSS) conducted in 2021, which included 7,218 individual samples. This study used COVID-19 vaccination as indicators for the health of interest, the key independent variable is Internet health information seeking. This study tried to analyze the impact of Internet health information seeking on COVID-19 vaccination using an OLS model and PSM method.

**Results:**

The results show that Internet health information seeking has a significant positive impact on COVID-19 vaccination. This result passed a series of robustness tests. The mechanism analysis indicated that compared to non-Internet health information seeking individuals, Internet health information seeking individuals could know the superior advantages of vaccination and the potential for immunization through this method. And individuals who use Internet to seeking health information are more likely to acknowledge the constructive impact of online information on health behavior. This helps to explain why Internet health information seeking individuals have a higher rate of COVID-19 vaccination.

**Conclusions:**

This study delves into the influence of Internet health information seeking on individual COVID-19 vaccination within the digital era. The outcomes underscore that Internet-mediated seeking vaccine information holds the potential to bolster individuals’ comprehension of vaccination benefits and foster increased acceptance of such information.

## Introduction

Vaccination is a safe and effective public health measure that helps protect individuals and social groups from disease [1–4]. Amid the COVID-19 pandemic, the significance of vaccination has been emphatically underscored. As the foremost and pivotal measure for preventing COVID-19 transmission, the COVID-19 pneumonia vaccine plays an instrumental role in the global response to this infectious respiratory disease. Its authorization by the World Health Organization (WHO) for emergency use on a global scale underscores its paramount importance. Clinical trials and real-world evidence have substantiated the vaccines’ effectiveness in providing robust protection against infection, severe illness, and mortality while maintaining a commendable safety profile. These vaccines are potent tools in humanity’s arsenal for effectively mitigating the transmission and proliferation of COVID-19 [5–10].

An investigation in the United Kingdom revealed that a single dose of any COVID-19 vaccine exhibited an efficacy of approximately 80% in averting COVID-19 hospital admissions and 85% in preventing mortality attributed to the disease [7]. Moreover, comprehensive protection, with an efficacy rate of 91%, was demonstrated under full vaccination [8]. Findings from an extensive empirical study encompassing 1.2 million participants in Israel further accentuate the vaccine’s efficacy, indicating a robust 94% effectiveness in guarding against symptomatic infections [9, 10].

However, COVID-19 vaccination coverage remains challenging in low- and middle-income countries and regions. According to statistics, as of April 2022, the global average COVID-19 vaccine full vaccination rate is 61%, of which the COVID-19 vaccination rate in some economically backward regions, such as South Africa, have a COVID-19 full vaccination rate of 32%, Iraq is 18%, and Afghanistan is only 13%. Therefore, increasing COVID-19 vaccination coverage in low-income areas at this stage remains an urgent issue [11].

China’s population ranks among the highest in the world [12]. Fearing that the population will suffer a catastrophe under the condition of underdeveloped medical infrastructure, China has been adhering to the Zero-COVID strategy for nearly three years until November 2022, effectively protecting the population from the epidemic [13]. However, highly infectious omega-clonal variants increase the economic and social costs of containment, seriously challenging this strategy. As the number of infections surges in November 2022, the public and the government are pushed to the brink, both psychologically and economically. The Chinese government finally ended the Zero-COVID policy. As of December 11, 2022, most cities will no longer require testing for SARS-CoV-2 in public places, and people infected with SARS-CoV-2 will no longer need to be quarantined [14]. Before the Zero-COVID policy ended, vaccinators routinely turned away people they suspected might be at risk of side effects, such as vaccinating people with pre-existing conditions such as diabetes, heart disease, stroke, kidney disease and cancer [15]. The definition of “contraindication” is broad and vague, leading to public distrust of vaccines. Therefore, after the end of the Zero-COVID policy in China, how to effectively eliminate the public misunderstanding of COVID-19 vaccination and increase the vaccination rate has become crucial.

Information asymmetry, that is, the lack of correct knowledge of the effect of vaccination against COVID-19, is the main reason individuals in low-income regions and countries refuse to receive the COVID-19 vaccine. Research on perceptions of vaccination around the world during and after the development of COVID-19 vaccines has also shown that personal perceptions of COVID-19 vaccines can affect vaccination behaviors, such as ignorance of the effectiveness of vaccination [16] and concerns about potential side effects [17], and underestimating the benefits of vaccination [18].

The relationship between proactive health information seeking and health behaviors is well documented [19]. Several observational studies have found that frequent exposure to health information is also associated with healthier behaviors, such as more regular physical activity and fruit and vegetable consumption, as well as fewer cigarettes and excessive alcohol consumption [20, 21]. With the spread of the Internet/modern information technology, using the Internet to search for health knowledge is very common, but it is worth exploring whether using Internet to seeking health information can promote health behaviors. On the one hand, such a wealth of health information on the Internet can be beneficial, as the plethora of ideas on the market ideally leads to greater awareness and engagement and promotes better awareness and the adoption of effective methods to prevent, detect, and treat health conditions [22], but on the other hand, Internet information is also often filled with myths or overtly false information that can adversely affect public beliefs and have adverse consequences for public health [23].

While there has been sevarl research on individual health behaviors, only a few studies have looked at the impact of Internet information on COVID-19 vaccination [24]. In this literature, the researchers discuss the effect of the percentage of COVID-19 vaccine-related news that is true or false on vaccination behavior. In this study, we choose from the perspective of individuals using the Internet for health information seeking, to explores the impact of individuals’ Internet health information seeking on their vaccination behavior, and what is the mechanism of such impact? Is there heterogeneity in this effect due to differences in individual characteristics? To provide solid empirical research evidence for the subsequent better use of the Internet to improve people’s health information, such as vaccination.

The present study aims to fill these gaps in the literature by examining the effect of Internet health information seeking to health information on an individual’s vaccine uptake based on data from the Chinese General Social Survey (CGSS2021). Specifically, we pursue three objectives. First, we describe the rate of an individual’s COVID-19 vaccination and examine the difference among subgroups based on Internet health information seeking status. Second, we estimate the impact of Internet health information seeking on an individual’s COVID-19 vaccination. At last, we explore possible channels that Internet health information seeking influences an individual’s COVID-19 vaccination. The remaining paper is structured as follows: We introduce the data and describe the variables. We then provide the econometric models and indemnification strategies, followed by the estimation results and discussion. Finally, we summarize the findings and suggest policy implications.

## Method

### Data

The data utilized in this research originates from the Chinese General Social Survey (CGSS) conducted in 2021. Initiated by the China Survey and Data Center of Renmin University of China, the CGSS is a large-scale continuous random sample survey project that commenced in 2003. It represents the earliest national, comprehensive, and continuous academic survey project in China, widely recognized as an authoritative database with an extensive number of survey samples, broad coverage, and diverse content. The CGSS 2021 database constitutes the most recent data released by the survey, comprising 8,148 valid samples, which systematically and comprehensively offer multi-level data on social, family, and individual aspects, adequately catering to the research requirements regarding Internet health information seeking and vaccination. Consequently, a total of 7,281 samples were retained for the study.

### Variables

In this study, COVID-19 vaccination was employed as indicator of the health of interest. Specifically, we constructed one dummy variable to measure whether an individual has been vaccinated against COVID-19 (= 1 if yes; = 0 if no). In the CGSS2021 questionnaire, the specific question about dependent variables was “Currently, whether you are vaccinated against COVID-19 or not”. We define individual has been vaccinated against COVID-19 if their answer is “YES”.

The key independent variable is Internet health information seeking, Specifically, we constructed one dummy variable to measure whether an individual has gotten information about vaccination on the Internet (= 1 if yes; = 0 if no). In the CGSS2021 questionnaire, the specific question about dependent variables was “In the past 12 months, whether to go online to search for information about vaccinations”. We define Internet health information seeking to receiving vaccine information if their answer is “YES.”

In addition to Internet health information seeking, an individual’s vaccination behavior may be influenced by many other factors. We control a set of variables in our empirical models to avoid bias from omitted variables. Control variables included demographic and family characteristics of the individual in the present study, including gender (male = 1), age (years), ethic (han = 1), educational attainment, family economic condition, marriage (married = 1), type of occupation( work at state-owned enterprise = 1), health status (scores improve with physical fitness: very unhealthy = 1, relatively unhealthy = 2, healthy = 3, relatively healthy = 4, very healthy = 5), and living area (rural = 1). Table 1 presents descriptive statistics for these key variables.

Finally, this study needs to explore the mechanism by which Internet health information seeking influences individuals’ vaccination behavior. Based on the existing literature, recognition of the benefits of vaccination is a key driver of vaccine uptake and the profound impact of attitudes to information on individual health behaviors. According to previous literature, we constructed the following three dummy variables: “Believed that the benefits of vaccination outweigh the disadvantages”, “Believed that vaccination gives immunity more than disease” and “Believed that Internet information has a positive impact on health behaviors”. The above three variables are constructed from the answers to the following three questions in the CGSS2021 questionnaire, which is “Whether you agree that the benefits of vaccination outweigh the disadvantages”, and “Do you agree that immunity through vaccination is better than disease”, and “Do you agree that over the past 12 months, the information on the Internet has had a positive impact on my health behaviors.”

### Statistics analysis

This study first summarized descriptive statistics of individual and family background characteristics. Moreover, group comparisons of these characteristics between Internet health information seeking and non-Internet health information seeking were performed by sample t-tests to examine any difference in means between the two groups of individuals.

Next, to analyze the main interest of this study, we employed the ordinary least squares (OLS) regression model to estimate the impact of Internet health information seeking on individual’s COVID-19 vaccination behavior.

1 $$ {{\text{Y}}_{\text{i}}} = \alpha+ {\beta _1}\,{\text{Internet}}\,{\text{health}}\,{\text{information}}\,{\text{seekin}}{{\text{g}}_{\text{i}}} + {\beta _2}{{\text{X}}_{\text{i}}} + {\varepsilon _{\text{i}}} $$

where ${\text{Y}}_{\text{i}}$ is a binary indicator for the COVID-19 vaccination behavior of individual i. where ${\text{I}\text{n}\text{t}\text{e}\text{r}\text{n}\text{e}\text{t} \text{h}\text{e}\text{a}\text{l}\text{t}\text{h} \text{i}\text{n}\text{f}\text{o}\text{r}\text{m}\text{a}\text{t}\text{i}\text{o}\text{n} \text{s}\text{e}\text{e}\text{k}\text{i}\text{n}\text{g}}_{\text{i}}$ is a dummy variable indicating whether the individual use Internet to seeking vaccine information. where ${\text{X}}_{\text{i}}$ represents a vector of baseline variables that would be correlated with individual’s COVID-19 vaccination behavior. These baseline variables include demographic factors (gender, age, ethic, educational attainment), family factors (family economic condition, marriage, health statue, and living area), and vaccination behavior-related factors (health statue). ${\varepsilon _{\text{i}}}$is a random error term.

In the regression models, we adjust standard errors for clustering at the individual level using the cluster-corrected Huber-White estimator. The Sidak adjustment method is used to correct the P-values of multiple comparisons. All analyses were performed using Stata 15.0 (Stata Corp., Texas, USA). All tests were two-sided, and *P* < 0.1 was considered statistically significant.

## Result

### COVID-19 vaccination and background characteristics

Table 1 presents summary statistics of these variables for all samples and separately by whether they were Internet health information seeking. In all samples, the COVID-19 vaccination rate was 73.1%, the proportion of males was 45.8%, the mean age was 51.3 years, 93% were Han Chinese, and most of the respondent’s marital status (75.1%) were married. In addition, the survey sample was in good health, with an overall sample self-reported health score of 3.47 points. The living area distribution of the overall sample shows that 46.1% of the respondents live in the eastern region, 33.3% live in the central region, and 20.6% live in the western region.

The Internet health information seeking group had a COVID-19 vaccination rate of 84.5%, while the non-Internet health information seeking group had a COVID-19 vaccination rate of 71.6%. This difference was statistically significant (*p* < 0.01). Additionally, there were statistically significant variations in the two groups distributions of age, education level of primary school and below, education level of high school and above, household wealth status of poor, household wealth status of rich, occupational type, and health (*p* < 0.01). The Internet health information seeking group is younger, more educated, wealthier regarding household assets, work in a state-owned enterprise, and healthier than the group not use Internet to seeking health information. There are no significant differences between the two groups regarding gender, ethnicity, marital status, and living area.

**Table 1:** Summary statistics of background characteristics

Variable, mean(SD)	Full sample	Non Internet health information seeking	Internet health information seeking	T-test
				Difference
	(1)	(2)	(3)	(2)-(3)
**Panel A: Outcome variables**				
1.COVID-19 vaccine(1 = yes, 0 = no)	0.731(0.443)	0.716(0.451)	0.845(0.362)	-0.129***
**Panel B: personal characters**				
2.Gender(1 = male, 0 = female)	0.458(0.498)	0.460(0.498)	0.444(0.497)	0.016
3.Age(years)	51.302(17.221)	52.499(17.151)	42.105(14.830)	10.394***
4.Ethic(1 = Han, 0 = other)	0.929(0.258)	0.929(0.257)	0.927(0.260)	0.002
5.Education				
Primary school and below	0.330(0.471)	0.358(0.480)	0.121(0.326)	0.237***
Middle school	0.287(0.452)	0.283(0.451)	0.316(0.465)	-0.033**
High school and above	0.382(0.486)	0.359(0.480)	0.563(0.496)	-0.205***
6.Household Wealth				
Poor	0.331(0.471)	0.343(0.475)	0.240(0.427)	0.103***
Average	0.317(0.465)	0.321(0.467)	0.288(0.453)	0.033*
Rich	0.352(0.478)	0.336(0.472)	0.473(0.500)	-0.137***
7.Work_type(1 = State-owned enterprise, 0 = No)	0.101(0.301)	0.095(0.293)	0.148(0.355)	-0.053***
8.Marriage(1 = yes, 0 = no)	0.751(0.433)	0.750(0.433)	0.761(0.426)	-0.011
9.Health(1 = Very unhealthy 2 = Relatively unhealthy 3 = Healthy 4 = Relatively healthy 5 = Very healthy)	3.470(1.085)	3.430(1.096)	3.777(0.946)	-0.347***
10.Living area				
Eastern China	0.461(0.498)	0.460(0.498)	0.468(0.499)	-0.008
Central China	0.333(0.471)	0.332(0.471)	0.340(0.474)	-0.008
Western China	0.206(0.405)	0.208(0.406)	0.192(0.394)	0.016
N	7281	6443	838	

Note: *** *p* < 0.01, ** *p* < 0.05

The correlation between the independent variables and the control variables and the result variables is analyzed, and the results are shown in Table 2. As can be seen from the results table, there is a positive correlation between independent variable internet health information seeking and dependent variable (correlation coefficient *r* = 0.093, *P* < 0.01). Other relevant control variables are also correlated with the outcome variables.

**Table 2:** Correlation coefficients

Variable	COVID-19 vaccine
1.Internet health information seeking	0.093***
2.Gender	-0.031***
3.Age	-0.298***
4.Ethic	-0.042***
5.Education	
Primary school and below	-0.130***
Middle school	0.026**
High school and above	0.102***
6.Household Wealth	
Poor	-0.075***
Average	0.053***
Rich	0.022*
7.Work_type	0.124***
8.Marriage	0.049***
9.Health	0.205***
10.Living area	
Eastern China	-0.133***
Central China	0.033***
Western China	0.125***

Note: *** *p* < 0.01, ** *p* < 0.05, * *p* < 0.1

### Effects of Internet health information seeking on individual’s COVID-19 vaccination

Table 3 shows the results that Internet health information seeking has a significant positive impact on COVID-19 vaccination. The average COVID-19 vaccination rate among individuals that non-Internet health information seeking was about 71.6% (Table 2, row 13). The COVID-19 vaccination rate among individuals that Internet health information seeking was 4.1% points higher (Table 2, row 1). The result is significant at the 1% level. Furthermore, individuals who are younger, belong to ethnic minorities, possess moderate household wealth, are married, maintain better health, and reside in the central and western regions of China exhibit elevated rates of COVID-19 vaccination. This correlation demonstrates statistical significance at the 1% level. Gender and middle-school education were also significantly associated with COVID-19 vaccination rates. Vaccination rates are higher among females and those with middle-school education. The result is significant at the 5% level.

**Table 3:** Effect of Internet_exposure on rates of Covid-19 vaccine (OLS with Sidak adjustment )

Variable	COVID-19 vaccine
1. Internet health information seeking	0.041***
	(0.015)
2. Gender	-0.025**
	(0.010)
3. Age	-0.006***
	(0.000)
4. Ethic	-0.064***
	(0.019)
5. Education	
Middle school	0.027**
	(0.013)
High school and above	0.007
	(0.015)
6. Household Wealth	
Average	0.065***
	(0.012)
Rich	-0.008
	(0.014)
7. Work_type	0.109***
	(0.017)
8. Marriage	0.088***
	(0.011)
9. Health	0.050***
	(0.005)
10. Living area	
Central china	0.078***
	(0.011)
Western china	0.180***
	(0.013)
11. Constant	0.782***
	(0.035)
12. Observations	7,281
13. R-squared	0.146
14. Mean without Community_support population	0.716

Note: Values in parentheses are robust standard error; *** *p* < 0.01, ** *p* < 0.05

### Robustness check

The results of this study thus far indicate a correlation between Internet health information seeking and COVID-19 vaccination rates. To ensure the robustness of our model’s results, we conduct rigorous validation using the propensity score matching method. Due to the fact that Internet health information seeking is not a random event and can be influenced by a variety of factors, such as age, studies have shown that the younger generation is more likely to use the Internet than the older generation [25], and the level of education is also an important influence, and people with a high level of education are more likely to be proficient in using the Internet [20], there is the possibility of bias in the estimates. This issue, if left unaddressed, could result in significant deviations in the distribution of relevant eigenvalues. Therefore, implementing the propensity score matching method becomes essential for accurately estimating the impact of Internet health information seeking. Given that the population of individuals without Internet health information seeking is seven times larger than those with Internet health information seeking, we employ the one-to-seven matching method. In addition, both caliper matching and kernel matching are employed to ensure the reliability of our analyses.

Table 4 shows the matching results. The ATT value derived using one-to-seven matching was 0.050 and significant at the 1% level, indicating that the percentage of vaccination was 5.0% higher with Internet health information seeking than without. The ATT values obtained using Caliper matching and Kernel matching were 0.045(*P* < 0.01) and 0.052 (*P* < 0.01), respectively, which were consistent with the results of one-to-seven matching, and this result verified the robustness of the regression analysis results.

Second, considering the impact of different occupations on COVID-19 vaccination behavior, we replaced our dependent variable with “COVID-19 vaccination intention (1 = Yes 0 = No)“(column 2). In the CGSS2021 questionnaire, the specific question about this variables was “Which of the following statements best suits your situation?”. We define intention to receiving vaccine if their answer is “I wanted to be vaccinated.”and the results were similar.

**Table 4:** Robustness check

Variable	COVID-19 vaccine	COVID-19 vaccine_want
Panel A: PSM		
1. One to seven matchingg	0.050***	
	(0.015)	
2. Caliper matching	0.045***	
	(0.013)	
3. Kernel matching	0.052***	
	(0.013)	
Panel B: Change outcome variable		
1. Internet health information seeking		0.030*
		(0.018)

Note: Values in parentheses are robust standard error; *** *p* < 0.01, *P < 0.1

### Mechanisms

In this subsection, we delve into potential mechanisms to gain a deeper comprehension of how disseminating vaccine health information through the Internet influences individuals’ vaccination behavior. Table 5 presents a tripartite breakdown of the pathways through which Internet health information seeking shapes vaccination patterns.

Drawing insights from existing literature, recognizing vaccination benefits is a pivotal driver of vaccine uptake. We embarked on an exploration of the link between Internet health information seeking and individuals’ perceptions regarding the merits of vaccination, encompassing notions such as the supremacy of vaccination benefits over drawbacks and the establishment of immunity through vaccination. As delineated in Table 5, columns 1 and 2, the outcomes revealed a heightened likelihood among Internet health information seeking individuals to acknowledge the superior advantages of vaccination and the potential for immunization through this method. This accurate understanding consequently serves as a catalyst propelling Internet health information seeking individuals towards vaccine adoption.

**Fig. 1:**
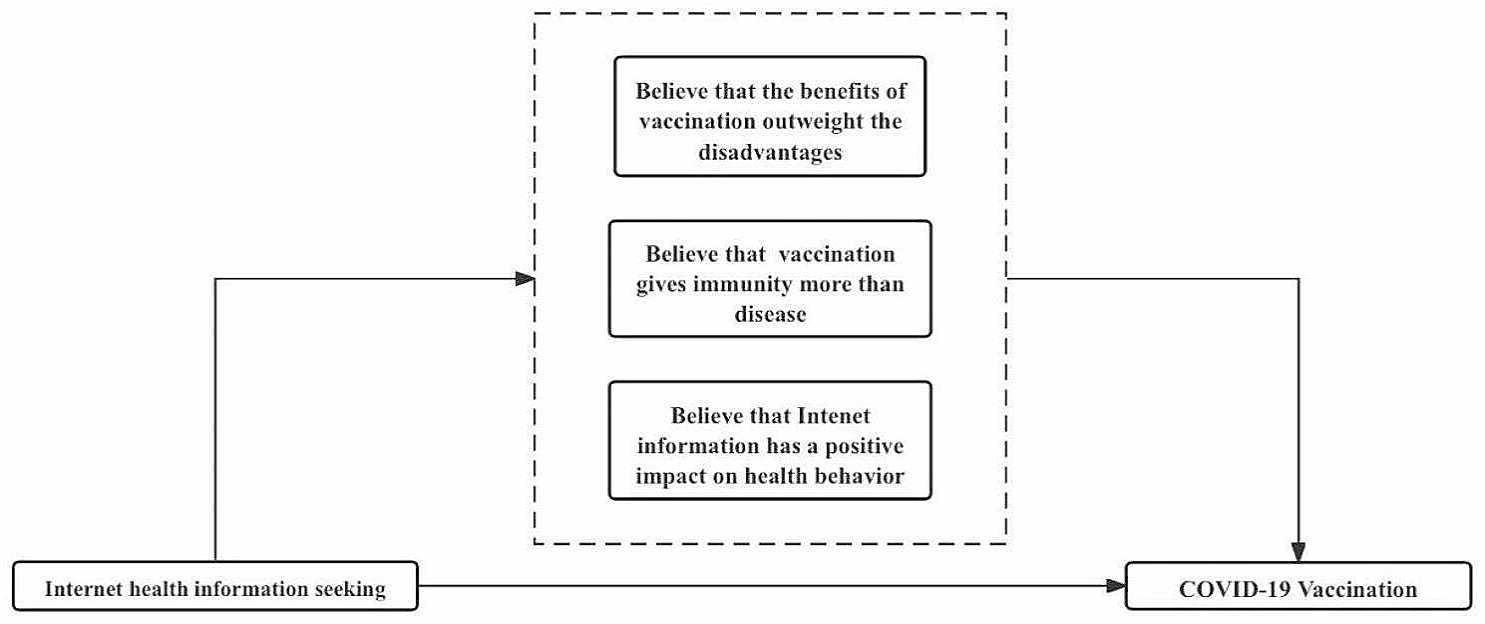
The mediating effects of cognitive factors

Approaching the matter from an alternative angle, empirical studies have demonstrated the profound influence of attitudes towards information on individual health behaviors. This investigation explored the impact of Internet health information seeking on individuals’ recognition of the positive effect of Internet-based information on health behaviors. As depicted in Table 5, column 3, the findings underscore a stronger propensity among Internet health information seeking individuals to acknowledge the constructive impact of online information on health behaviors. This acknowledgment engenders a greater acceptance of the veracity of Internet information, thereby fostering the execution of health-conscious behaviors. A visual representation of this impact mechanism is illustrated in Fig. 1.

**Table 5:** Mechanism analysis

Variable	Believe that the benefits of vaccination outweigh the disadvantages	Believed that vaccination gives immunity more than disease	Believe that internet information has a positive impact on health behaviors
1. Internet health information seeking	0.709***	0.708***	0.683***
	(0.015)	(0.015)	(0.012)
2. Control variable	YES	YES	YES
3. Mean in no Internet health information seeking	0.193	0.204	0.102
4. N	7281	7281	7281

Note: Values in parentheses are robust standard error; *** *p* < 0.01

### Heterogeneous analysis

We extend our analysis to encompass the heterogeneous effects of Internet health information seeking by incorporating interaction terms. As evidenced in Table 6, our investigation reveals limited indications of heterogeneous effects across various demographic and household attributes within our surveyed cohort. These attributes encompass gender, education level, household wealth, health status, and geographical location. Notably, we did identify specific subgroups in which Internet health information seeking seems to have a discernible impact on vaccination likelihood.

Upon closer examination, our findings indicate that older age, Han Chinese ethnicity, and unmarried status correlate with an augmented probability of vaccination due to Internet health information seeking, as indicated in Table 6. This pattern can be ascribed to distinct factors that operate within these subgroups. For instance, older individuals might exhibit heightened receptivity to health-related information, rendering them more responsive to the effects of Internet health information seeking on vaccination rates, as detailed in Table 6, row 2. Furthermore, the influence of Internet health information seeking on vaccination rates appears to be more pronounced among the unmarried demographic. This can potentially be attributed to the relatively higher availability of time and energy among unmarried individuals, in contrast to their married counterparts who often contend with familial responsibilities. As elaborated in Table 6, row 7, those without marital commitments might allocate more resources to safeguarding their physical well-being, making them particularly responsive to the impact of Internet-based health information on their vaccination choices.

**Table 6:** Heterogeneous effect

Variable	COVID-19 vaccine
1. Internet health information seeking*Gender	0.028
	(0.003)
2. Internet health information seeking*Age	0.003***
	(0.001)
3. Internet health information seeking*Ethic	0.120**
	(0.058)
4. Education	
Internet health information seeking*Middle school	-0.022
	(0.049)
Internet health information seeking*High school and above	-0.049
	(0.047)
5. Household Wealth	
Internet health information seeking*Average	-0.021
	(0.041)
Internet health information seeking*Rich	0.016
	(0.038)
6. Internet health information seeking*Work_type	-0.074
	(0.045)
7. Internet health information seeking*Marriage	-0.085**
	(0.035)
8. Internet health information seeking*Health	-0.019
	(0.016)
9. Living area	
Internet health information seeking*Central china	0.026
	(0.034)
Internet health information seeking*Western china	-0.031
	(0.041)

Note: Values in parentheses are robust standard error; *** *p* < 0.01, ** *p* < 0.05

## Discussion

Few published quantitative studies have measured the effect of Internet health information seeking on individual vaccination against COVID-19. Based on a nationally representative dataset in China, our empirical analysis provides evidence and contributes to a more comprehensive understanding of the impact of Internet health information seeking on individual vaccination against COVID-19. Taking particular vaccination against COVID-19 as an indicator variable, our research results show that Internet health information seeking has a positive impact on individual COVID-19 vaccination behavior.

Our research revealed that Internet health information seeking has a considerable positive impact on individual COVID-19 vaccination behavior. This finding aligns with prior studies that have explored the influence of Internet health information seeking on individual health-related actions. For instance, earlier investigations have illustrated that encountering Internet information contributes to better behaviors concerning smoking and alcohol consumption among individuals [26]. Additionally, descriptive studies have pointed out that individuals actively searching for vaccine-related information on the Internet tend to exhibit higher vaccination rates [27].

Furthermore, we have delved into the underlying mechanisms to attain a more profound comprehension of how exposure to vaccine-related health information on the Internet influences an individual’s vaccination behavior. The study’s outcomes unveiled that the elevated COVID-19 vaccination rates among individuals who accessed Internet-based vaccine information could potentially be attributed to the alteration in their perceptions regarding vaccination benefits. These individuals exhibited an increased recognition of the idea that the benefits of vaccination outweigh any potential drawbacks, while also grasping the concept of immunity development through vaccination.

According to the Knowledge-Attitude-Practice health behavior theory, acquiring health information can effectively improve the individual’s awareness of the benefits of healthy behavior, enhance the individual’s behavioral motivation, and thus improve the individual’s behavior [28, 29]]. Information seeking can facilitate access to new information and may reinforce normative beliefs and perceptions of healthy behaviors [30]. Prior research has also highlighted that individuals’ misconceptions about vaccine information constitute a significant hurdle to non-vaccination. Therefore, when individuals come into contact with Internet-based information [31], they gain access to accurate information, rectifying information asymmetry and enhancing their vaccination behavior.

On the other hand, an individual’s attitude towards information also affects an individual’s health behavior [27]. Our findings suggest the impact of Internet health information seeking on individuals’ awareness of the positive effects of Internet-based information on health behaviors. This study explores the impact of Internet health information seeking on individuals’ awareness of the positive effects of Internet-based information on health behaviors. The findings suggest that Internet health information seeking individuals are more likely to recognize the constructive effects of online information on health behaviors. This recognition increases the acceptance of the authenticity of information on the Internet, thereby promoting the implementation of health-conscious behaviors.

Heterogeneity analysis results showed that Internet health information seeking had a greater impact on the vaccination behavior of the older, Han, and unmarried groups. The possible reason is that older people grew up before the popularization of the Internet, and they rely more on traditional media, social circles, and traditional medical institutions to obtain health information. Therefore, once they start using the Internet, they may be more sensitive to new ways of obtaining information and thus have a greater impact [32]. Han people may be more digitally literate to use the Internet, and more likely to acquire, interpret, and apply health information, so that Internet health information seeking has a greater impact on their health behaviors [33]. As for the impact of Internet health information seeking on vaccination rates, it seems to be more pronounced among unmarried people. This may be due to the fact that unmarried people have relatively more time and energy, while married people tend to have family responsibilities. At the same time, those without marital commitment may allocate more resources to safeguarding their own physical health, making them particularly sensitive to the impact of Internet-based health information on their vaccination choices [34].

This study has significant advantages and is a valuable addition to the literature. First, by providing causal insights compared with descriptive analysis, it theoretically reveals the impact of individuals’ Internet health information search on their intention and behavior of vaccination, providing a basis for individual health decision-making theory in the Internet age. Secondly, the results of this study show that Internet information search improves people’s cognition of the value of vaccination behavior, helps to understand the impact of individuals’ access to health information from the Internet on health decision-making, and provides insights for the field of behavioral economics and health decision-making. In the end, our results are critical for how future Internet port-based interventions can be designed to reduce intervention costs and efficiency.

The study also had several limitations. First, we did not investigate the impact of Internet health information seeking frequency on COVID-19 vaccination due to data limitations. Moreover, it’s important to note that our results could have been influenced by self-report bias, given that vaccination status relied on self-reported data. Additionally, our dataset from CGSS did not include data on the severity of the outbreak (i.e. average daily number of new cases, average number of deaths) and individuals’ attitudes towards COVID-19 vaccination, which should be taken into account in future studies.

## Conclusions

The relentless invasion of the COVID-19 pandemic has revolutionized the spring of 2020. Vaccination against COVID-19 stands as a crucial tool in containing its spread, with nations striving to heighten vaccine coverage rates. A potent avenue for enhancing individual vaccination behavior is exposure to accurate vaccine information. Thus, this study delves into the influence of Internet vaccine information seeking on individual COVID-19 vaccination within the digital era. The outcomes underscore that Internet-mediated exposure to vaccine information holds the potential to bolster individuals’ comprehension of vaccination benefits and foster increased acceptance of such information. Consequently, this positively impacts COVID-19 vaccination behavior, fortifying the overall effort.

## Data Availability

The datasets supporting the conclusions of this study are available from the corresponding author upon reasonable request.
